# On the Role of Attention in Working Memory for Response Selection in Task Switching

**DOI:** 10.5334/joc.69

**Published:** 2019-08-08

**Authors:** Darryl W. Schneider

**Affiliations:** 1 Department of Psychological Sciences, Purdue University, US

**Keywords:** Attention, Working memory, Cognitive control, Executive functions

## Abstract

Oberauer’s (2019) analysis and related research on how working memory and attention are linked can provide insight regarding how responses are selected in task-switching situations. One mechanism for response selection—the mediated route—categorizes stimuli with respect to both tasks, then activates responses based on instructed category–response associations. The author discusses two proposals for how these associations are represented in working memory, both of which seem consistent with the idea that attention selects or prioritizes the relevant-task associations, enabling accurate response selection. A broader implication of the representation and operation of the mediated route is that nominal task switching might reflect concurrent multitasking within the cognitive system (constrained by attention in working memory), raising the issue of how task switching should be characterized.

Working memory (WM) and attention are critical for controlling thought and action, especially when switching tasks. In this commentary, I first discuss ideas concerning how responses are selected in task-switching situations, then consider the role of attention in WM for controlling response selection, drawing on Oberauer’s (2019) analysis and related research.

## Response Selection in Task Switching

People can switch tasks, but not as easily as they can repeat tasks (Kiesel et al., 2010). Task switching is difficult because it sometimes involves ambiguity: a stimulus might require different responses for different tasks, raising uncertainty about which response is appropriate. Consider the tasks of categorizing the referents of word stimuli as living/nonliving or small/large, responding with a left keypress for living or small, and a right keypress for nonliving or large. Incongruent stimuli (e.g., *bear*) are mapped to different responses across tasks, whereas congruent stimuli (e.g., *bee*) are mapped to the same response. The ambiguity of incongruent stimuli is reflected in response congruency effects: worse performance for incongruent than for congruent stimuli (e.g., Kiesel, Wendt, & Peters, 2007; Meiran & Kessler, 2008; Schneider, 2015).

Mediated and nonmediated routes for response selection can explain response congruency effects (Kiesel et al., 2007; Meiran & Kessler, 2008; Schneider, 2015, 2018). The mediated route categorizes stimuli with respect to both tasks, then activates responses based on instructed category–response associations. The nonmediated route bypasses categorization and activates responses based on learned stimulus–response associations. Response selection is impaired for incongruent stimuli because conflicting responses are activated, whereas it is facilitated for congruent stimuli because a unique response is activated, producing response congruency effects.

The two routes are not mutually exclusive and there is evidence supporting each of them. Evidence for the mediated route includes findings of response congruency effects when categorization was the sole mechanism for response selection because the nonmediated route was rendered nonfunctional by using nonrepeated stimuli (Schneider, 2015, 2018), nonpracticed stimulus–response associations (Liefooghe, Wenke, & De Houwer, 2012), or distractors never presented as target stimuli (Reisenauer & Dreisbach, 2013). Evidence for the nonmediated route includes findings of inverted response congruency effects when category–response associations were reversed, indicating persisting use of learned stimulus–response associations (Schneider & Logan, 2015; Wendt & Kiesel, 2008).

## The Role of Attention in WM for Response Selection

The mediated and nonmediated routes both depend on long-term memory for associations: stimulus–category associations in the mediated route (e.g., *bee* is living and small) and stimulus–response associations in the nonmediated route that are formed during practice. However, the mediated route depends on WM for instructed category–response associations (e.g., living and small categories mapped to a left keypress). How are these associations represented in WM?

Within the embedded-components model of WM (Cowan, 1999; Oberauer, 2002, 2009; Oberauer & Hein, 2012), one possibility is that category–response associations are available in the activated part of long-term memory (Meiran & Kessler, 2008). Considering that only one task is to be performed on each stimulus in typical task-switching experiments, the subset of category–response associations for the relevant task needs to be prioritized, which is possible if they are held in the embedded region of direct access. Drawing on Oberauer’s (2019) suggestion that “WM is attention to memory representations,” one could argue that attention selects which associated representations in activated long-term memory are held in the direct-access region. From this perspective, category–response associations for both the relevant and irrelevant tasks are in WM, but the mediated route enables accurate response selection—even for incongruent stimuli—because the most relevant associations have more attentional weight. However, the irrelevant associations in activated long-term memory can prime response selection, yielding response congruency effects. When switching tasks, attention would select the newly relevant associations for the direct-access region based on a task representation that guides attention, such as a goal (Rubinstein, Meyer, & Evans, 2001) or mediator (Logan & Schneider, 2006).

Oberauer, Souza, Druey, and Gade (2013) provided an alternative conceptualization of how the mediated route can be represented in WM to produce response congruency effects. In their connectionist model, relevant-task category–response associations are represented as temporary bindings in what they called the bridge of procedural WM, which is analogous to the direct-access region. However, response congruency effects in the model reflect the influence of residual bindings for the irrelevant task in the bridge, not priming from irrelevant-task associations in activated long-term memory. The import of this difference is debatable. The bindings in the bridge are represented by gain-modulating units, whose activation is updated to implement the relevant task set based on input from units activated by the task cue. When switching tasks, the matrix of bindings is reconfigured for the newly relevant task. If one interprets reconfiguring the binding matrix as analogous to changing attentional weights for relevant- and irrelevant-task associations, then the model is functionally similar to my earlier sketch of the mediated route within the embedded-components model, albeit with a blurred distinction between the bridge (or direct-access region) and activated long-term memory. Moreover, an attentional interpretation of how bindings are activated in the bridge of Oberauer et al.’s model seems consistent with Oberauer’s (2019) conclusion that the “selection of information to be held in WM is a form of controlled attention.”

The idea that irrelevant-task associations remain active, even though attention selects or prioritizes the relevant-task associations for the mediated route in WM, raises the issue of how task switching should be characterized. As discussed previously (Schneider, 2015, 2018), when the mediated route determines response selection, it implies that a form of dual-task processing underlies behavior, despite overt performance of a single task on each trial of typical task-switching experiments. It follows that nominal task switching might reflect concurrent multitasking within the cognitive system, constrained by attention in WM that facilitates selection of the relevant-task response on most trials. If this view has merit, then further consideration of Oberauer’s (2019) conceptual analysis of how attention and WM are linked will be important for continued theoretical progress in task switching.
